# Health-related Quality of Life in Children and Adolescents After Successful Treatment of Chronic Hepatitis C With Sofosbuvir/Velpatasvir: One-year Outcomes

**DOI:** 10.1097/INF.0000000000004675

**Published:** 2024-12-20

**Authors:** Maria Pokorska-Śpiewak, Anna Dobrzeniecka, Ewa Talarek, Małgorzata Aniszewska, Magdalena Pluta, Magdalena Marczyńska, Giuseppe Indolfi

**Affiliations:** From the * Department of Children’s Infectious Diseases, Medical University of Warsaw, Warsaw, Poland; † Department of Pediatric Infectious Diseases, Regional Hospital of Infectious Diseases in Warsaw, Warsaw, Poland; ‡ Department of Neurofarba, Meyer Children’s University of Florence, Florence, Italy; § Meyer Children’s Hospital IRCCS, Florence, Italy.

**Keywords:** children, health-related quality of life, hepatitis C virus, sofosbuvir/velpatasvir

## Abstract

**Background and aims:**

The aim of this study was to assess the health-related quality of life (HRQL) of children with chronic hepatitis C (CHC) at 1 year after the effective treatment with sofosbuvir/velpatasvir (SOF/VEL).

**Methods:**

All 50 patients treated for CHC with a fixed dose SOF/VEL in the noncommercial, nonrandomized, open-label PANDAA-PED study achieved sustained virologic response at 12 weeks after the end of treatment. Evaluation of HRQL at 1-year posttreatment was compared with the baseline (before the treatment) assessment. KIDSCREEN-27 questionnaires, which included 5 dimensions of HRQL, for child self-reporting and parent proxy reporting were used. The normal range for the population was set to T values of 40–60 points. Child–parent agreement was analyzed using the intraclass correlation coefficient (ICC).

**Results:**

Mean T values were within the normal range for all HRQL dimensions. A significant improvement in “autonomy & parent relation” in children’s self-assessment (from 48.3 to 51.5, *P* = 0.03) was observed. In parent proxy assessment, a significant decrease occurred in “school” dimension (from 49.5 to 45.8, *P* = 0.03), which was not revealed at 3-month posttreatment. Older age was associated with worse HRQL scores in all dimensions. Evaluation of the ICC for child self-reports versus parent proxy reports revealed poor-to-moderate agreement for most single measures, lower than at 3-month posttreatment analysis.

**Conclusions:**

This is the first study to present the long-term influence of treatment with direct-acting antivirals on patient-reported outcomes in children. At 1 year after effective treatment with SOF/VEL, an improvement in some areas of children’s well-being was revealed, which may indicate also some patient-reported outcomes benefits of direct-acting antiviral therapy. Despite the improvement in the child self-report of “autonomy & parent relation,” there was a more pronounced discrepancy between children self-reports and parents proxy reports in all dimensions of HRQL. Older patients’ age correlated with worse HRQL assessment. If this finding is mediated by the duration of hepatitis C virus infection, it would support recommendation for the treatment of younger children.

Hepatitis C virus (HCV) infection has been considered not only a liver disease but a systemic condition leading to multiple extrahepatic manifestations, which may negatively influence both clinical and patient-reported outcomes (PROs).^1^ It was shown that patients with chronic hepatitis C (CHC) may develop a diminished health-related quality of life (HRQL) even before advanced stage of liver disease. However, effective treatment of HCV has been associated with significant improvement in PROs.^1^ In adult patients who achieved sustained virologic response (SVR) after treatment with direct-acting antivirals (DAAs), significant increases in well-being as determined by PRO scores were shown, whereas decrease in PRO scores was observed in participants treated ineffectively.^2^

New interferon-free regimens based on DAAs were proven to be safe and effective also in pediatric patients and are now recommended for treatment in adolescents and children as young as 3 years of age.^3,4^ There are only limited data on HRQL in pediatric patients with CHC.^5–9^ HCV infection in children may have a detrimental effect on their HRQL, leading to increased caregiver stress, strain on the family system, and poorer health status, including impaired psychosocial, social, and cognitive functioning.^5,7–9^ In addition, a significant reduction in a wide range of intelligence and memory scores among children with CHC was reported.^10^ On the other hand, treatment of HCV in adolescents with sofosbuvir-based regimens or with glecaprevir/pibrentasvir (GLE/PIB) was shown to improve patients’ HRQL.^5,6,11^

Recently, we reported a significant proportion of children with CHC having decreased scores in various dimensions of HRQL (in 7%–24% of child self-reports and in 14%–36% of parent proxy reports) before treatment.^12^ These observations are concordant with other studies that analyzed HRQL in children with chronic liver diseases, showing significantly impaired HRQL in these patients compared with healthy controls.^8,9,13^ In a study on 114 treatment-naïve children with CHC aged 11 ± 3 years, 18% of patients had impaired cognitive functioning; 13% had somatic problems, depression, and/or anxiety; and 9% presented with aggressive behavior or social problems.^7^ In our cohort, the effect of HCV on HRQL was more pronounced in older patients, and thus, we concluded that treatment of younger children should be indicated to prevent them from experiencing decreased HRQL due to ongoing HCV infection. In addition, effective treatment with sofosbuvir/velpatasvir (SOF/VEL) in this cohort led to an improvement in some areas of well-being at 3-month posttreatment.^12^ In this second part of this study, we aimed to analyze the HRQL in children and adolescents at 1 year after successful treatment with SOF/VEL to establish long-term outcomes. In addition, we investigated the agreement between children’s and their caregivers’ assessments.

## METHODS

### Study Design

This is the second part of the recently described analysis on the HRQL assessment in children aged 6–18 years treated with SOF/VEL in the PANDAA-PED study (“Treatment of chronic hepatitis C in children 6–18 years of age using a pangenotypic direct-acting antiviral (sofosbuvir/velpatsvir)”).^12,14^ In brief, PANDAA-PED is a noncommercial, nonrandomized, open-label study founded by the Medical Research Agency, Warsaw, Poland (grant number 2019/ABM/01/00014), which enabled treatment of 50 pediatric patients with CHC for 12 weeks with a fixed dose of SOF/VEL adjusted to the patients’ weight. Treatment efficacy was defined as SVR12, that is, undetectable HCV RNA using a real-time polymerase chain reaction method at 12-week posttreatment. The full PANDAA-PED study design was described previously.^14^ Recruitment for this study was initiated in January 2022, and all the children completed their SVR12 evaluation before December 31, 2022. In this part of the study, we included all participants that completed the 1-year posttreatment visit.

### HRQL Assessment

Patients’ HRQL was evaluated using the KIDSCREEN-27 questionnaire (The KIDSCREEN Group, 2004; EC Grant Number: QLG-CT-2000-00751, KIDSCREEN-27, Child and Adolescent Version and Parent Version in Polish).^15,16^ This instrument was found to have excellent cross-cultural comparative validity.^15,17^ The questionnaire consists of 27 items divided into the 5 health-related dimensions, described in detail in our previous paper.^12^ The parent proxy version of the questionnaire has the same structure and it asks the caregivers to assess their children’s HRQL from their own perspective. In the case of children aged below 8 years, the assessment was made only by their parents/guardians, as the child and adolescent version of the KIDSCREEN-27 is suitable only for children aged at least 8 years.

The KIDSCREEN-27 questionnaires were completed by patients and their parents/caregivers simultaneously at baseline (before the treatment), then at 12 weeks after the end of the 12-week therapy (results of this assessment were described previously^12^), and finally, at 1-year posttreatment to establish the long-term effects of SOF/VEL on patients’ HRQL, which were described in this part of the analysis.

To make the interpretation of the results more applicable, the obtained Rasch scores for each of the 5 dimensions were further translated into T values with a mean of 50% and 95% confidence interval (CI). The population norm was set at T-distribution between 40 and 60 points with higher T values indicating higher HRQL.^16^

### Statistical Analysis

Continuous data were presented as means (95% CI), and they were compared using Student’s *t*-test for paired samples or repeated measures analysis of variance. To determine the aGreement between the assessment performed by children and their parents/caregivers, the intraclass correlation coefficient (ICC) was calculated. ICC was interpreted according to the Portney and Watkins criteria as follows: <0.75, poor-to-moderate agreement; 0.75–0.90, good agreement; and >0.90, reasonable agreement for clinical measurements.^18^ To analyze the correlation between children’s age and the HRQL assessment, a correlation coefficient r (95% CI) was calculated. A 2-sided *P* value <0.05 was considered to indicate significance. All statistical analyses were performed using MedCalc Statistical Software version 22.018 (MedCalc, Ostend, Belgium, https://www.medcalc.org).

### Ethical Statement

The local ethics committee of the Medical University of Warsaw approved this study (approval numbers KB/136/2020, September 14, 2020; and KB/30/A2021, April 19, 2021). The PANDAA-PED study was performed in accordance with the ethical standards of the 1964 Declaration of Helsinki and its later amendments. Written informed consent was collected from all the patients and/or their parents/guardians before their enrollment in the study.

## RESULTS

### Patients

**FIGURE 1.**
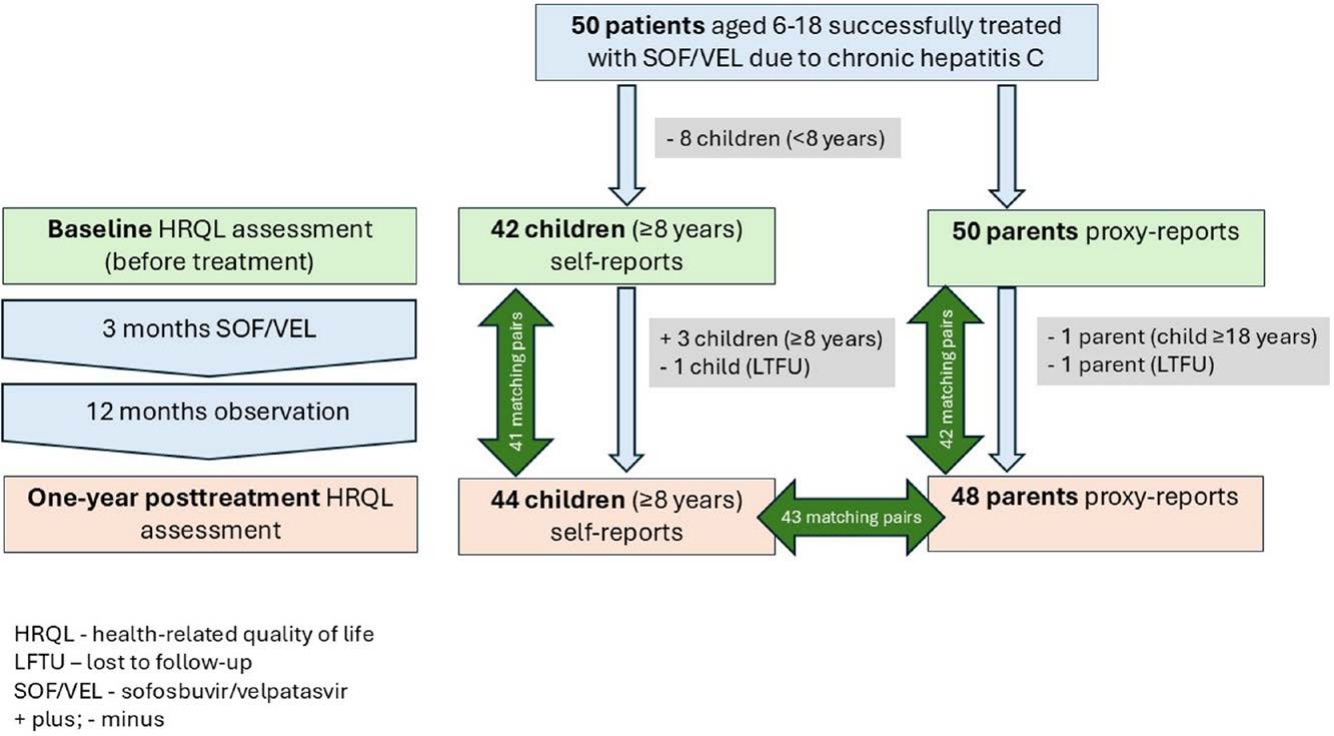
Flowchart of participants and their parents who made HRQL assessments at baseline and 12-month posttreatment.

All 50 participants treated with SOF/VEL achieved SVR12. Among them, 49 patients completed the control visit at 1-year posttreatment. One 13-year-old girl was lost to follow-up after the 3-month posttreatment visit. The flowchart presenting the number of patients and HRQL assessments at baseline and 1-year posttreatment, including numbers of analyzed matching pairs, was presented in Figure 1.

### Pre- and Posttreatment HRQL Evaluation

**FIGURE 2.**
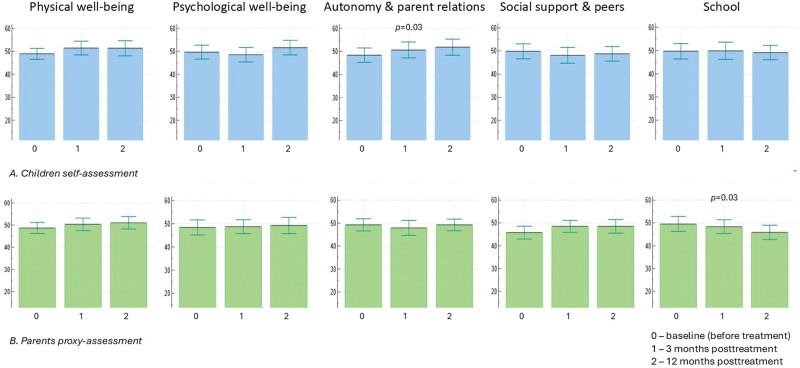
Changes in the assessment of 5 dimensions of HRQL by children and their parents at 3-month and 12-month posttreatment compared to baseline (before the treatment). Bars represent mean T values and horizontal lines indicate 95% confidence intervals. Data were compared using ANOVA (repeated measures analysis of variance). *P*-values were indicated only if they were <0.05.

Mean T values for all dimensions of HRQL were within the normal ranges (Table 1). Children self-report analysis revealed a significant increase in the mean T values at 1-year posttreatment compared with baseline in dimension “autonomy & parent relation” (51.5 vs. 48.3, *P* = 0.03). An insignificant increase was observed for “physical well-being” and “psychological well-being,” whereas in dimensions “social support & peers” and “school” insignificant small decrease was shown (Table 1). In the case of parent proxy assessments, a significant decrease occurred in the “school” dimension from 49.5 to 45.8 (*P* = 0.03). In addition, there was a trend toward higher mean T values for “physical well-being” and “social support and peers” dimensions (Table 1). Figure 2 shows changes in the assessment of 5 dimensions of HRQL by children and their parents at 3- and 12-month posttreatment compared with baseline (before the treatment). The analysis of variance testing confirmed a significant increase in the child self-assessment of “autonomy & parent relations” dimension after SOF/VEL treatment and a significant decrease in “school” dimension in parent proxy evaluation (Fig. 2).

**TABLE 1.** HRQL before and 1 year after the end of treatment with SOF/VEL (mean T values for 5 dimensions of the KIDSCREEN-27 instrument)

Dimension	Child self-reports (n=41)			Parent proxy reports (n=48)		
	Baseline	12-month posttreatment	*P* value	Baseline	12-month posttreatment	*P* value
Physical well-being	48.9 (46.5–51.3)	51.3 (48.0–54.6)	0.10	48.5 (46.1–51.0)	50.9 (48.2–53.7)	0.06
Psychological well-being	49.6 (46.5–52.6)	51.5 (48.4–54.7)	0.14	48.3 (45.1–51.5)	49.2 (45.7–52.7)	0.57
Autonomy & parent relation	48.3 (45.2–51.5)	51.8 (48.3–55.3)	**0.03**	49.2 (46.6–51.9)	49.2 (46.7–51.7)	0.99
Social support & peers	49.8 (46.6–53.1)	48.7 (45.5–51.9)	0.54	45.7 (42.9–48.5)	48.5 (45.5–51.5)	0.07
School	49.7 (46.3–53.0)	49.1 (46.0–52.1)	0.58	49.5 (46.2–52.8)	45.8 (42.6–49.3)	**0.03**

Data are presented as the mean T values (95% confidence intervals), and they were compared using paired sample *t*-test.

### Comparison of Child–Parent HRQL Assessment

Comparative analysis of the child self-responses and parents proxy assessment at 1-year posttreatment revealed lower mean T scores for all dimensions of the KIDSCREEN-27 questionnaire among parents/caregivers responses (Table 2). There were significant differences in the assessment of “psychological well-being” and “school” dimensions. In addition, the ICC evaluation for child self-reports versus parent proxy reports revealed poor-to-moderate agreement for all single measures and poor-to-good agreement for average measures (Table 3), which indicates that parent proxy reports may not be used interchangeably with children’s self-reports. Interestingly, ICCs were even lower compared with the analysis at 3-month posttreatement.^12^

**TABLE 2.** Comparison of HRQL assessment between child self-reports and parents proxy reports at 1-year posttreatment (43 matching pairs)

Dimension	Children	Parents	*P* value
Physical well-being	51.9 (48.6–55.1)	50.3 (47.5–53.2)	0.19
Psychological well-being	51.6 (48.5–54.7)	48.6 (44.8–52.4)	**0.02**
Autonomy & parent relation	52.1 (48.6–55.6)	49.1 (46.4–51.8)	0.09
Social support & peers	48.8 (45.8–51.9)	48.7 (45.4–52.0)	0.94
School	49.3 (46.3–52.3)	45.8 (42.3–49.2)	**0.01**

Data are presented as the mean T values (95% confidence intervals).

**TABLE 3.** Intraclass correlation coefficient (ICC) calculated for child self-reports vs. parent proxy-reports at 1-year posttreatment

Dimension	Single measures	Average measures
Physical well-being	0.71 (0.52–0.83)	0.83 (0.69–0.90)
Psychological well-being	0.70 (0.52–0.83)	0.83 (0.68–0.90)
Autonomy & parent relation	0.37 (0.09–0.60)	0.54 (0.17–0.75)
Social support & peers	0.28 (0.008–0.54)	0.44 (0.01–0.70)
School	0.66 (0.45–0.80)	0.79 (0.62–0.89)

Data are presented as intraclass correlation coefficients (ICCs) (95% confidence intervals). Interpretation: <0.75, poor-to-moderate agreement; 0.75–0.90, good agreement; and >0.90, reasonable agreement for clinical measurements.

### The Influence of Age on HRQL

In most cases, for both child self-reports and parent proxy reports, the children’s age correlated negatively with mean T scores (Table 4). For the following dimensions, “physical well-being,” “psychological well-being,” and “school,” the negative influence of age on the HRQL assessment was significant (Table 4).

**TABLE 4.** The influence of patient age on subsequent HRQL dimensions of the KIDSCREEN-27 according to child self-reports and parent proxy reports at 1-year posttreatment

Dimension	Children	Parents
Physical well-being	−0.39 (−0.61 to −0.11); *P* = 0.006	−0.44 (−0.54 to −0.18); *P* = 0.001
Psychologic well-being	−0.35 (−0.59 to −0.06); *P* = 0.01	−0.30 (−0.53 to −0.01); *P* = 0.03
Autonomy & parent relation	0.0 (−0.29 to 0.28); *P* = 0.97	−0.18 (−0.44 to 0.01); *P* = 0.21
Social support & peers	−0.13 (−0.41 to 0.16); *P* = 0.37	−0.19 (−0.45 to 0.09); *P* = 0.18
School	−0.27 (−0.52 to 0.02); *P* = 0.07	−0.30 (−0.54 to −0.01); *P* = 0.03

Data are presented as correlation coefficient r (95% confidence intervals); *P*-value.

## DISCUSSION

SOF/VEL combination is effective and safe for the treatment of children as young as 3 years of age, and thus, it is among the preferable pangenotypic DAAs recommended for CHC treatment in children and adolescents.^3,4,14,19^ In a largest study presenting treatment effects of SOF/VEL in 216 children aged 3–17 years with HCV infection, there were, however, 2 cases of serious psychiatric adverse events of suicidal ideation with 1 suicidal attempt and bipolar disorder.^19^ Both cases occurred in teenagers with complex medical history and were probably unrelated to the study drug.

Thus, it seems essential to analyze the influence of DAA treatment not only on liver disease but also on PROs, including HRQL. Our previous report of the PANDAA-PED study presented the influence of SOF/VEL therapy on HRQL in 50 patients aged 6–18 years at 3-month posttreatment.^12^ We documented a significant increase in self-reported physical well-being and a trend toward higher social support and peers scores in parent evaluations. In addition, there was an increasing proportion of children who estimated that their physical well-being improved. These results supported the hypothesis that successful treatment of HCV led not only to clinical improvement but also to benefits for PROs also in children and adolescents. Similar observations were made by other authors analyzing the effects of DAAs on children’s HRQL up to 24-week posttreatment. The influence of the following DAAs on HRQL has been previously studied in patients aged at least 12 years: SOF/ledipasvir, SOF/ribavirin (RBV), and GLE/PIB.^5,6,11^ In a study analyzing the effects of SOF/ledipasvir therapy in 100 adolescents, parent proxy reports of HRQL improved at the end of treatment, whereas self-reported scores did not change compared with baseline. In addition, at 12-week posttreatment, the parent reports remained higher, and patient self-reports of emotional functioning were also higher than those at baseline. All observed improvements were sustained up to the 24-week follow-up.^5^ In another cohort of 50 patients treated with SOF/RBV, some improvement was reported after achieving SVR based on patient self-reported social functioning at posttreatment week 12 and parent-reported school functioning by week 24 posttreatment.^6^ In the DORA study on GLE/PIB treatment among the 44 adolescents, improvements in patient-reported HRQL, physical health summary score, and psychosocial health summary score were observed.^11^ All these studies, however, analyzed mainly adolescents >12 years of age and did not exceed the 24-week posttreatment period. PANDAA-PED study included children as young as 6 years of age and – to our knowledge – analyzed the longest available duration of the follow-up.

The current part of the study aimed to analyze whether the obtained effects on the HRQL maintain for the longer period after the achievement of SVR12. Thus, we have repeated the HRQL assessment at 12-month posttreatment and compared the results with baseline pretreatment values. We found a significant increase in the mean T values at 1-year posttreatment compared with baseline in child self-reports in dimension “autonomy & parent relation.” In the parent proxy assessments, a significant decrease occurred in the “school” dimension. In contrast, there was a trend toward higher mean T values for “physical well-being” and “social support and peers” dimensions. The decrease in the assessment of “school” dimension did not occur at 3-month posttreatment. It does not necessarily reflect the treatment outcomes, but rather may be a result of the greater school workload for over 1-year-old children. However, Younossi et al.,^5^ who analyzed HRQL in 50 adolescents with HCV infection treated with SOF/RBV showed that school functioning appeared most affected by HCV infection among the assessed dimensions. In this cohort, patients reported difficulties in the school functioning, and their scores in this dimension were significantly lower than those in healthy controls.^5^ Similar observations were made in another group of 100 adolescents with CHC, whose HRQL scores related to physical and social functioning were normal, but school functioning scores were significantly lower than those of the normal youth population.^6^

Another finding of our study, presented at 1-year and 3-month posttreatments, was the influence of the patients’ older age on worse assessment of most HRQL dimensions in both child self-reports and parents’ proxy reports. This observation is consistent with reports of other authors who demonstrated significantly higher scores of HRQL among patients with chronic liver disease in younger age groups.^13^ This indicates that the effect of chronic liver disease on psychosocial health is more pronounced in adolescents, which should be considered as another indication for starting treatment for CHC as soon as possible. On the other hand, there is no evidence that this age-related worsening HRQL was only due to ongoing viremia or hepatic inflammation. The influence of comorbidities, intrauterine drug exposure, and genetic or social challenges, which could affect HRQL and may be more apparent with age, were not analyzed in this study. However, most participants (94%) of the study group had been infected vertically, and thus, the duration of HCV infection was equal to their age in most cases.

In the first part of our study, we also analyzed the child–parent agreement of evaluation of the child’s HRQL, and we revealed a significant proportion of discrepancies, reaching from 35% to 60% depending on the studied dimension of HRQL, with poor-to-moderate ICCs in most cases. A similar observation was made at 1-year posttreatment. The parents’ perception of the child’s HRQL was worse than child’s self-report in all studied dimensions. The ICCs were in most cases even lower compared with the values obtained at 3-month posttreatment. This is an interesting observation taking into consideration the significant improvement in “autonomy & parent relation” in children self-reports at 1-year posttreatment compared with the baseline. However, other authors also reported that parents tended to assess HRQL scores lower than their adolescent children.^5,6,8^ Since most children with CHC became infected by their mothers, the caregivers are often afraid that their children’s health was worsen and they had concerns about their children’s behavioral problems and health status which make them limit their family activities.^7^

This is the first study presenting the long-term influence of the anti-HCV treatment on PROs in children. However, several limitations should be highlighted. First, we included a relatively small number of study participants. Not all children were able to make an assessment, as KIDSCREEN-27 questionnaire is suitable only for children aged at least 8 years. In addition, data on the psychological and social status of patients’ parents and guardians were not obtained.

In conclusion, the results of this part of the PANDAA-PED study indicate that at 1 year after effective treatment with SOF/VEL an improvement in some areas of children’s well-being was revealed, which may indicate also some PROs benefits of DAA therapy. However, patients’ school functioning in parents’ proxy reports decreased. In addition, despite the improvement in the child self-report of “autonomy & parent relation,” there was a more pronounced discrepancy between children self-reports and parents proxy reports in all dimensions of HRQL. Older patients’ age correlated with worse HRQL assessment. If this finding is mediated by the duration of HCV infection, it would support recommendation for the treatment of younger children.
